# Impaired visual working memory and reduced connectivity in undergraduates with a *history* of mild traumatic brain injury

**DOI:** 10.1038/s41598-021-80995-1

**Published:** 2021-02-02

**Authors:** Hector Arciniega, Jorja Shires, Sarah Furlong, Alexandrea Kilgore-Gomez, Adelle Cerreta, Nicholas G. Murray, Marian E. Berryhill

**Affiliations:** 1 Department of Psychology, Programs in Cognitive and Brain Sciences, and Integrative Neuroscience, University of Nevada, 1664 N. Virginia St., MS 296, Reno, NV 89557 USA; 2 Psychiatry Neuroimaging Laboratory, Brigham and Women’s Hospital, Harvard Medical School, Boston, MA 02215 USA; 3 School of Community Health Sciences, University of Nevada, Reno, 89557 USA; 4 Department of Psychology and Neuroscience, University of North Carolina at Chapel Hill, Chapel Hill, NC 27599 USA

**Keywords:** Psychology, Human behaviour, Neuroscience, Learning and memory, Working memory

## Abstract

Mild traumatic brain injury (mTBI), or concussion, accounts for 85% of all TBIs. Yet survivors anticipate full cognitive recovery within several months of injury, if not sooner, dependent upon the specific outcome/measure. Recovery is variable and deficits in executive function, e.g., working memory (WM) can persist years post-mTBI. We tested whether cognitive deficits persist in otherwise healthy undergraduates, as a conservative indicator for mTBI survivors at large. We collected WM performance (change detection, n-back tasks) using various stimuli (shapes, locations, letters; aurally presented numbers and letters), and wide-ranging cognitive assessments (e.g., RBANS). We replicated the observation of a general *visual* WM deficit, with preserved *auditory* WM. Surprisingly, visual WM deficits were equivalent in participants with a history of mTBI (mean 4.3 years post-injury) and in undergraduates with recent sports-related mTBI (mean 17 days post-injury). In seeking the underlying mechanism of these behavioral deficits, we collected resting state fMRI (rsfMRI) and EEG (rsEEG). RsfMRI revealed significantly reduced connectivity within WM-relevant networks (default mode, central executive, dorsal attention, salience), whereas rsEEG identified no differences (modularity, global efficiency, local efficiency). In summary, otherwise healthy current *undergraduates* with a history of mTBI present behavioral deficits with evidence of persistent disconnection long after full recovery is expected.

## Introduction

Mild traumatic brain injury (mTBI), or *concussion* accounts for nearly 235,000 hospitalizations each year^1–4^, with 85% of cases categorized as mTBI^5,6^ in the USA *alone*. Many mTBI cases go untreated because people do not seek medical care^7^. Treatment involves initial rest followed by a gradual return to normal activities. Full recovery of cognitive function is expected within months without further rehabilitation^8^. *But does cognition fully recover after mTBI?*

Certainly, in moderate and severe TBI cognitive impairment persists^9–11^. In acute mTBI (0–3 days post injury) impairments include deficits in processing speed^12,13^, attention^14–16^, and episodic memory^17^. However, the data on cognitive outcomes long after mTBI (> 3 months) are mixed. For example, recent reviews reported no lasting cognitive deficits^18–20^, whereas athletes and veterans at > 1-year post-injury have lasting executive dysfunction^21–40^, and impaired working memory (WM)^41,42^. In these populations, effects may be heightened by blast injury^43^, and/or repeated head injury^44^. Reports in civilian mTBIs do not note cognitive deficits but do identify impaired peripheral vision, tandem gait, and psychosocial ability^45,46^. As many reconsider the consequences of brain trauma, we recently reported that healthy *undergraduates* self-reporting a *history* of mTBI (hmTBI, > 4 years post-injury) performed significantly worse than their peers on visual WM tasks (e.g., 3-color patches × 900 ms delay)^47,48^. *We replicated this observation in six experiments testing 135 hmTBI participants* while manipulating encoding, maintenance durations, retrieval demands, and presence of feedback^47,48^. The undergraduates reported mTBIs from typical childhood experiences (e.g., bike accidents, falling off the monkey bars), as well as sports. Meta-analysis of our work from the six experiments suggests that number of mTBIs, time since injury, and etiology did not predict visual WM performance, however, loss of consciousness *did.* Surprisingly, loss of consciousness predicted less WM impairment, we speculate that perhaps those who reported loss of consciousness sought medical treatment^48^. Nonetheless, there was a consistent, significant visual WM deficit in a young, healthy population who is *not* seeking or receiving treatment. Visual WM is an important executive function that is needed to integrate visual experience across eye movements, meaning deficits might interfere with the seamless representation of the external world.

Given that roughly 50% of mTBIs are undiagnosed or are not managed appropriately makes post injury care difficult to manage^49^. This is vitally important as initial presentation to a concussion specialty clinic within a week of injury tends to result in faster recovery compared to athletes evaluated 2–3 weeks post-injury^50,51^. If no injury assessment occurs, specific managements strategies to aid in recovery may not be implemented, which could lead to longer recovery time. Protracted recovery may lead to reorganization of functional networks, for example due to chronic exposure to pain^52^.

Why is visual WM impaired so long after mTBI? Lingering tissue damage can arise from shearing forces^53–58^ that disrupt neural connections^59,60^ in several WM relevant networks including the default mode network (DMN)^61,62^. Connectivity measurements identify altered default mode network (DMN) activity 10 years post mTBI^63^. DMN connectivity is also associated with processing speed^64^, and visuospatial task performance^65,66^. Several other networks demonstrate altered activity post-mTBI^67–69^, including the central executive network (CEN)^70,71^, dorsal attentional network (DAN)^72–74^, and salience network (SN)^75,76^.

It could be that visual WM is the only area where hmTBI participants are impaired, but this seems improbable. Here, we had several goals. First, to test whether our observation of a visual WM deficit extended to other visual WM tasks, and other visual stimuli, and whether group-level impairment would extend to auditory WM. We also wanted to test cognition more broadly to see if there were other areas of deficit in this undergraduate population. Second, to compare lasting WM deficit with initial effects, we included a group of undergraduates who had recently experienced an mTBI. Third, we tested whether the cause of performance deficits was due to altered network-level activity. In short, our goal was to understand the breadth of cognitive deficits in the hmTBI group, whether WM performance significantly improves post-mTBI, and to identify neural mechanisms underlying observed deficits. Importantly, if hmTBI alters cognitive outcomes in undergraduates—it can serve as a bellwether for the impact of mTBI in the general population. Experiments 1A-B tested the breadth of WM deficits in undergraduates with a hmTBI by including two tasks (3-back, change detection) and three kinds of stimuli (locations, shapes, letters) Experiment 2 compared cognitive performance in undergraduates with a hmTBI (no persistent symptoms, > 3 months post-mTBI) to those with a recent sports related-mTBI (SR-mTBI: 4 days–3 months post-injury); see Table 1. Experiment 3 probed connectivity (rsEEG, rsfMRI) to link behavior with underlying neural mechanisms. We hypothesized a pattern of generally impaired WM performance in the hmTBI group. We predicted a specific deficit in WM rather than a general cognitive deficit across all cognitive domains. We anticipated significantly worse performance in the SR-mTBI group consistent with the typical timeline of recovery over time. Additionally, we predicted the hmTBI group would demonstrate reduced connectivity following mTBI, in the networks selected for their involvement in WM (Table 3).

**Table 1:** Demographics for all experiments.

Experiment	Group	Age (SD)	# (# F)	# TBI	Range #	Time (SD)	Range time
**Experiment 1A**	hmTBI	20.9 (3.2)	25 (16)	1.9 (1.5)	1–7	3.9 y (6)	6 mo–25 y
	Control	22.2 (2.9)	25 (17)				
**Experiment 1B**	hmTBI	20.5 (2.53)	30 (22)	2.7 (2.1)	1–9	3.4 y (3.2)	7 mo–sss11.6 y
	Control	20.9 (2.33)	33 (23)				
**Experiment 2**	hmTBI	22.2 (3.3)	25 (11)	3.6 (2.6)	1–12	4.3 y (3.7)	5 m–12.2 y
	SR-mTBI	20.3 (2.0)	21 (15)	2.2 (1.5)	1–6	17 days (19.3)	3–80 days
	Control	25.1 (4.8)	25 (15)				
**Experiment 3**	hmTBI	22.3 (3.4)	23 (10)	3.6 (2.7)	1–12	4.2 y (3.6)	5 mo–12.2 y
	Control	24 (5.5)	23 (11)				

Separate cohorts were tested in each experiment. The mean number of mTBIs and the Time (in years) since the most recent mTBI, averaged across participants.

*#* number of mTBIs, *F* female, *hmTBI* history of mTBI group, *mo* months, *Range #* range of number of mTBIs, *Range Time* range of time since last mTBI, *recent SR-mTBI* recent sport related mTBI, *SD* standard deviation in years, *y* years.

## Results

**Figure 1:**
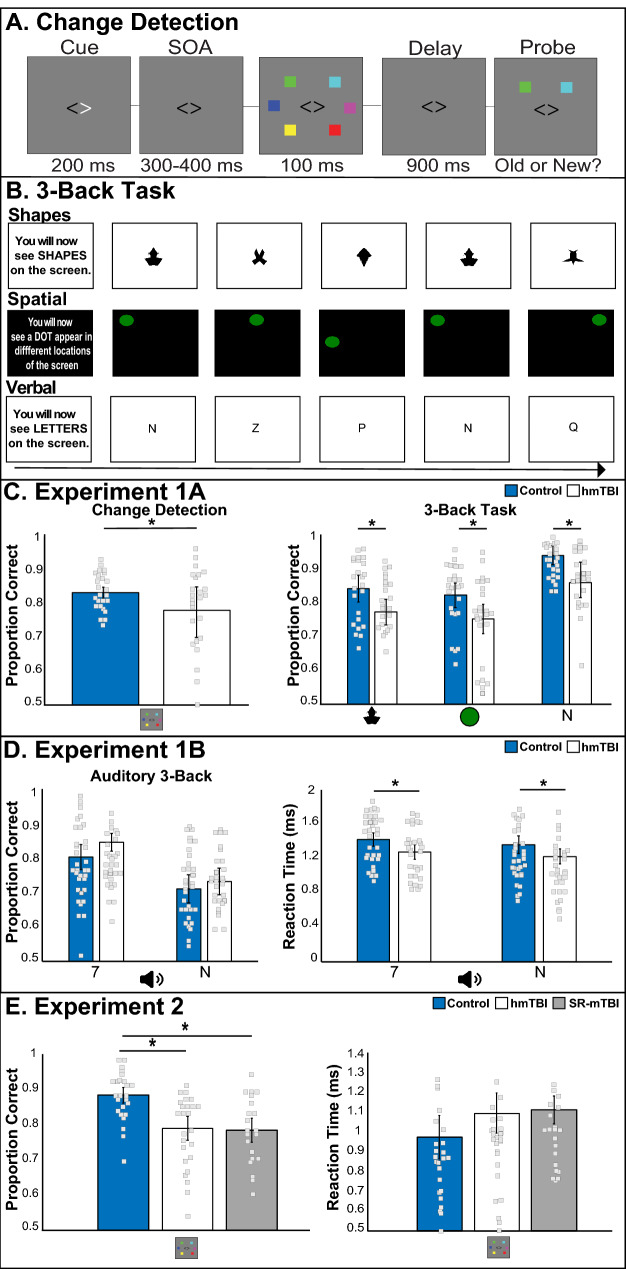
WM tasks and behavioral results. (**A**) Change detection and (**B**) 3-back task showing the shapes, spatial locations, and verbal stimuli used in Experiment 1A. Behavioral Results for Experiment 1A visual change detection and 3-back tasks (**C**). Those with a hmTBI performed significantly less accurately than controls across tasks and stimuli. (**D**). Experiment 1B used aurally presented digits and letters and observed no accuracy difference between groups but did reveal a significant speeding in the hmTBI group. (**E**) Behavioral results showing accuracy, but no reaction time deficit in both hmTBI and SR-mTBI compared to controls, in Experiment 2. *p < 0.05 and error bars represent 95% confidence intervals. *hmTBI* history of mTBI, *SR-mTBI* sports related mTBI.

The first two behavioral experiments tested WM in undergraduates with a hmTBI more broadly by including two WM tasks (change detection, 3-back) and three tasks (shapes, spatial, letters); Experiment 1A. We included two sensory modalities (vision, audition) to see if WM deficits were constrained to one modality or were supramodal; Fig. 1B. We first replicated the pattern of impaired change detection accuracy in those with a hmTBI. An independent sample t-test indicated that accuracy measures in the change detection task revealed that there significantly less information maintained in visual WM in the hmTBI (t(48) = 2.25, p = 0.03, d = 0.58; Fig. 1C); without effecting reaction times (t(48) = 0.27, p = 0.78, observed β = 0.92, n.s.). Performance on the 3-back task was examined with a repeated measures ANOVA across the two groups (control, hmTBI) and the three task types (shapes, spatial, letters). The analysis revealed a significant main effect of group (F_1,48_ = 9.62, p = 0.004, η^2^_p_ = 0.16; Fig. 1C; reaction time: p = 0.30, η^2^_p_ = 0.003, n.s.). Accuracy was superior for the letter stimuli (F_1,48_ = 44.4, p < 0.0001, η^2^_p_ = 0.39). Reaction times were faster responding to the shapes (F_1,48_ = 6.8, p = 0.002, η^2^_p_ = 0.12). There was no group × stimulus interaction (p = 0.73, observed β = 0.1). Experiment 1A confirmed a general *visual* WM deficit in undergraduates with a hmTBI. This raises the question of whether WM deficits are restricted to the visual domain or whether they extend to other sensory domains, such as auditory WM.

We then tested whether WM deficits were process-general by probing *auditory* WM in new cohorts (Exp. 1B). Participants completed an auditory 3-back task with blocks of digits and letters. Performance on the 3-back task was examined with a repeated measures ANOVA across the two groups (control, hmTBI) and the two auditory stimulus types (numbers, letters). Importantly, there was no main effect of group and no interaction on accuracy (ps > 0.4, observed β = 0.05). Accuracy was significantly higher during the digit trials (F_1,61_ = 64.6, p < 0.00001, η^2^_p_ = 0.51). Unexpectedly, reaction times in the hmTBI group were significantly *faster* than controls (F_1,61_ = 5.7, p = 0.02, η^2^_p_ = 0.08; Fig. 1D), and significantly faster for letter trials (F_1,61_ = 4.72, p = 0.03, η^2^_p_ = 0.07). No other comparisons approached significance (all ps > 0.15). These findings suggest that auditory WM was intact, and even more rapid, in those with h mTBI.

To compare results between experiment 1A and 1B we conducted repeated measures ANOVA with group (control, hmTBI) and experiment (1A, 1B) as factors for both the accuracy and reaction times of the letters task. There was a significant main effect of experiment (F_(1,109)_ = 80, p < 0.00001, η^2^_p_ = 0.4), where participants had higher accuracy scores in the visual WM task. Additionally, there was a significant interaction of group × experiment (F_(1,103)_ = 4.2, p = 0.04, η^2^_p_ = 0.04) indicating that control participants performance was higher across experiment. No other comparisons reached significance (all ps > 0.4, observed β = 0.14). The reaction times indicated a main effect of experiment (F_(1,109)_ = 77.9, p < 0.00001, η^2^_p_ = 0.42) where hmTBI participants had faster reaction times across experiments and a borderline effect of group (F_(1,109)_ = 2.9, p = 0.08, η^2^_p_ = 0.02) where hmTBI had faster reaction times. No other comparisons reached significance (all ps > 0.25, observed β = 0.2).

To test cognitive performance across domains we replicated the change detection WM test and conducted neuropsychological assessments of attention, episodic memory and learning in new participants in Experiment 2. We recruited a third group, athletes with a recent SR-mTBI to clarify the effect of recovery time on post-mTBI WM performance. This third group was necessary to begin to capture the amount of continued recovery to visual WM that could be anticipated over time. Finally, we collected resting state EEG connectivity in search of a biomarker sensitive to the visual WM impairment.

Change detection accuracy showed that compared to controls, both mTBI groups were impaired (F_(2,68)_ = 13.65, p < 0.00001, η^2^_p_ = 0.28; see Fig. 1E), but not different from each other (p = 0.82, observed β = 0.7). There were no significant differences in reaction times (F_(2,68)_ = 1.1, p = 0.3, η^2^_p_ = 0.03; see Fig. 1E). The neuropsychological tests revealed that both mTBI groups showed low index scores for immediate and delayed memory, consistent with the impaired learning and delayed recall observed on a test of verbal learning (CVLT; see Table 2). A measure of executive function identified a significant group difference (TMT-B: one-way ANOVA: F_(2,68)_ = 8.17, p = 0.001, η^2^_p_ = 0.19) with significantly slower performance in the hmTBI group (mean: 59.3 s, SD: 18.5, p = 0.0004; control mean: 41.7 s, SD: 10.3), but only a trend in the recent SR-mTBI group (mean: 50.2 s, SD: 16.3, p = 0.15). No group differences emerged in the sustained attention task (PVT: F_(2,68)_ = 1.3, p = 0.27, η^2^_p_ = 0.04). These data show preserved attention, visuospatial and recognition memory ability in the mTBI groups.

**Table 2:** Neuropsychological assessment data.

	hmTBI	SR-mTBI
	Mean (SD)	Mean (SD)
**RBANS indexes**		
*Immediate memory*	*88.7 (15.2)*	*88.7 (11.4)*
Visuospatial/constructional	100 (16.4)	100.2 (13.9)
Language	96 (16.4)	94.6 (15.8)
Attention	98.3 (16)	95.1 (20.9)
Delayed memory	91 (14.4)	*84 (18.3)*
Sum of index scores	472.6 (51.6)	459 (45.5)
Total scale	92.4 (12.8)	88.6 (11.2)
**CVLT-short**		
Trial 1: Free recall correct	− 0.92 (0.69)	− 1.2 (0.6)
Trial 2: Free recall correct	− 1.21 (0.69)	− 1.62 (0.5)
Trial 3: Free recall correct	***− 2 (0.5)***	***− 2.02 (0.5)***
Trial 4: Free recall correct	***− 2.24 (0.63)***	***− 2.4 (0.7)***
Trials 1–4 free recall total correct (T Score)	42 (9.4)	37 (7.5)
Short-delay free recall correct	− 1.72 (0.6)	− 1.8 (0.6)
Long-delay free recall correct	***− 2.38 (0.74)***	***− 2.3 (0.8)***
Long-delay cued recall correct	***− 2.64 (0.8)***	***− 2.6 (0.7)***
Free recall intrusions	0.06 (0.7)	0.2 (0.8)
Cued recall intrusions	− 0.3 (0.3)	− 0.3 (0.5)
Total intrusions	− 0.24 (0.7)	0.02 (0.7)
Total repetitions	− 0.92 (0.5)	− 0.8 (0.6)
Long-delay yes/no recognition hits	***− 4.92 (0.2)***	***− 4.95 (0.1)***
Long-delay yes/no recognition false positives	− 0.26 (0.56)	0.07 (0.6)
Long-delay forced-choice recognition accuracy	0.98 (0.04)	0.97 (0.05)

All RBANS index score values were between 90 and 109 and normal. CVLT-Short standardized scores > 1.96 are considered impaired (bold italicized).

*hmTBI* history of mTBI, *SR-mTBI* sports related mTBI.

We were interested in testing whether rsEEG connectivity measures would be sensitive to the visual WM deficit in the hmTBI group. Identifying a reliable biomarker would benefit recovery assessment and could serve as a potential target for continued rehabilitation. RsEEG data in the theta band were evaluated using measures of modularity, global efficiency and local efficiency. The theta band is linked to WM performance^77,78^. *Modularity* reflects the integration (global communication) or segregation (local processing) of within-network connections. *Global efficiency* measures information transfers across node-pairs to clarify integration. *Local efficiency* measures segregation of information transfer between neighboring electrodes. Data were compared using a mixed model ANOVA with the between-subject factor of group (control, hmTBI, SR-mTBI), and the within-subject factor of network cost (10%, 15%, 20%, 25%, 30%) to ensure results were not due to specific threshold values. There were no significant main effects of group across any measure (modularity: p = 1, global efficiency: p > 0.4, local efficiency: p > 0.5). For each measure, there was the expected main effect of network cost (all ps < 0.00001), as increasing network cost increases the threshold of the values. There were no interactions of group and network cost (all ps > 0.4). The rsEEG data found no evidence of altered connectivity in the hmTBI group at any network cost model.

**Figure 2:**
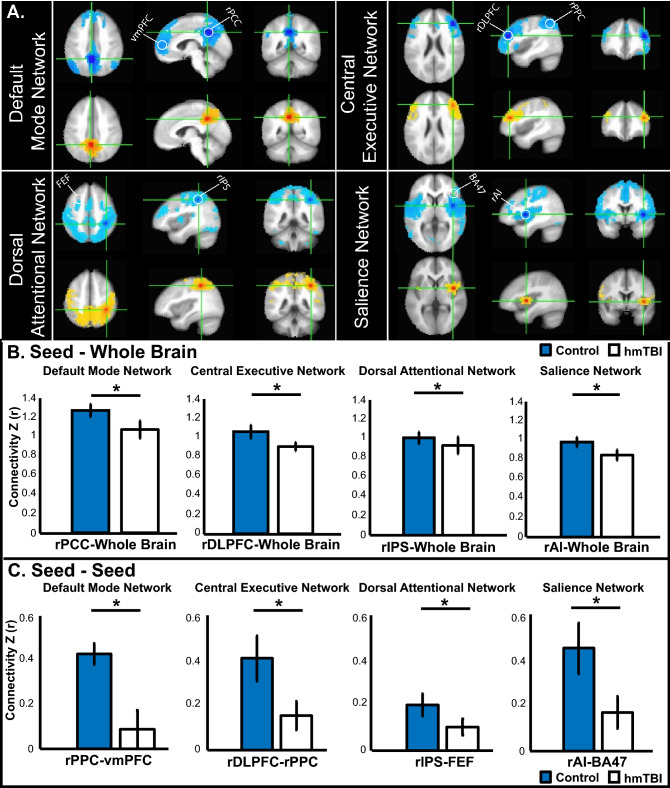
RsfMRI reveals weaker connectivity in participants with hmTBI. (**A**) DMN, CEN, DAN, and SN functional whole brain connectivity results show weaker connectivity in those with a hmTBI (yellow) compared to controls (blue). White circles identify seed locations. (**B**) Bar plots indicate z-score seed analysis based on seed-whole brain, indicating greater seed-whole brain connectivity in the control group. (**C**) Bar plots indicating z-score seed analysis showing connectivity between our seed-seed locations indicating greater connectivity in the control group. Error bars represent 95% confidence intervals. *hmTBI* history of mTBI, *SR-mTBI* sports related mTBI.

Next, we examined rsfMRI data from the hmTBI group who provided rsEEG (Exp. 2) to test for weakened connectivity. Using one seed region per network, we evaluated functional connectivity between our primary seed location and the whole brain (rPCC-Whole Brain, rDLPFC-Whole Brain, rIPS-Whole Brain, rAI-Whole Brain). Next, we selected a second seed location within each network and calculated connectivity from seed to seed (rPCC-vmPFC, rDLPFC-rPPC, rIPS-FEF, rAI-BA47). The first approach sampled whole brain connectivity, whereas the second approach probed within network connectivity. As shown by our independent sample t-test the hmTBI group had reduced *seed*—*whole brain* connectivity across all seeds (DMN: rPCC-Whole Brain: t (44) = − 3.4, p = 0.001 (hmTBI mean: 1.05, SD: 0.22; control mean: 1.24, SD: 0.15), rPCC-vmPFC: t (44) = − 6, p < 0.00001 (hmTBI mean: 0.12, SD: 0.2; control mean: 0.43, SD: 0.12); CEN: rDLPFC-Whole Brain: t (44) = − 2.1, p = 0.04 (hmTBI mean: 0.9, SD: 0.2; control mean: 1.05, SD: 0.17), rDLPFC-rPPC: t (44) = − 4.2, p < 0.00013 (hmTBI mean: 0.16, SD: 0.16; control mean: 0.43, SD: 0.26); DAN: rIPS-Whole Brain: t (44) = − 2.4, p = 0.02 (hmTBI mean: 0.93, SD: 0.17; control mean: 1.01, SD: 0.23), rIPS-FEF: t (44) = −3.4, p ≤ 0.003 (hmTBI mean: 0.1, SD: 0.08; control mean: 0.2, SD: 0.1). SN: rAI-Whole Brain t (44) = 3.9, p = 0.0003 (hmTBI mean: 1.05, SD: 0.22; control mean: 0.94, SD: 0.12), rAI-BA 47 t (44) = − 2.9, p < 0.006 (hmTBI mean: 0.05, SD: 0.07; control mean: 0.11, SD: 0.09); see Fig. 2A–C. The results indicate there is weaker network connectivity in the hmTBI between our seed locations and the whole brain and even in our more conservative approach of seed-seed connectivity.

Finally, to understand whether hmTBI alters the relationship between visual WM accuracy and connectivity, we conducted a Pearson correlation. There was a borderline effect in DMN (rPCC-Whole Brain: r = 0.37, p = 0.08), but no other evidence of significant correlations (DMN: rPPC-vmPFC: r = 0.07, p = 0.74); CEN: rDLPFC-Whole Brain: r = − 0.14, p = 0.5, rDLPFC-rPPC: r = − 0.05, p = 0.8; DAN: rIPS-Whole Brain: r = − 0.28, p = 0.2, rIPS-FEF: r = − 0.15, p = 0.5; SN: rAI-Whole Brain: r = − 0.28, p = 0.2, rAI-BA 47: r = 0.22, p = 0.3.

## Discussion

The consequences of mTBI can persist for years. To date, relatively few studies examine the long-term consequences of mTBI in civilian populations. Research typically tests military veterans with a history of blast injury or elite athletes, both at higher risk for repeated head injury. We adopted a different approach by testing otherwise healthy undergraduates with a hmTBI who were past the typical recovery stage. Findings in this population likely underestimate effects in the general population. These data support the existing literature that suggests mTBI can have long-term cognitive effects. Our data suggest that undergraduates with a history of show the traces of an mTBI ~ 3.8 years post-mTBI and that these effects are not different from student athletes who are only ~ 17 days post-mTBI.

We replicated our previous finding showing visual WM deficits in those with a hmTBI, who were well over 4 years post-injury. Unexpectedly, this visual WM deficit was equivalent to performance in those with a recent SR-mTBI. In other words—time may not heal this deficit. Surprisingly, *auditory* WM was well preserved as our findings suggest no differences in accuracy between the groups (control, hmTBI) and even faster reaction times in the hmTBI group. This is important because neuropsychological tests, such as the RBANS and the CVLT rely on auditory testing. Consequently, modality specific deficits may be obscured with auditory testing. Indeed, the cognitive battery revealed generally high function, as would be expected from an undergraduate sample. The exception was poor performance in immediate and delayed memory tasks in the hmTBI group. Connectivity analyses identify reductions across WM-relevant networks in the hmTBI group; see Fig. 2A–C. Even in otherwise healthy undergraduates there are lingering traces of an mTBI. An alternative hypothesis is that low WM capacity individuals experience mTBI more often than high WM capacity individuals, a hypothesis requiring prospective testing and long-term follow-up.

We speculated that longer white matter tracts, such as those associated with visual WM, would be more susceptible to heterogeneous trauma. This view is challenged by the overlap between network activity associated with auditory and visual WM^79^, and the observation of superior auditory WM in the hmTBI group. Future work including visual and auditory WM performance paired with connectivity measures is needed to understand how mTBI alters brain and behavior.

MTBI in the general population falls under the radar as large investigations examine mTBI in specific populations, such as American football players and veterans^33,80,81^. Yet, the current investigation may be targeting the 14% of mTBI patients seen privately and the estimated 25% who receive no medical attention^82^. MTBI can be consequential with detectible effects *years* after injury. To our knowledge, there are no ongoing therapeutic interventions designed to rehabilitate cognitive deficits in the hmTBI population. The functional connectivity data suggest that reduced connectivity persists after a mTBI and should be a target for future interventions.

This work has several important limitations. The unexpected observation that auditory WM was superior in the hmTBI group deserves additional research including both visual and auditory WM tasks. Secondly, the observation of weaker connectivity in the hmTBI population would benefit from a much larger sample so that the individual differences could be teased apart. However, we did attempt to identify relationships between accuracy and connectivity, but our sample size is too small to begin to draw strong conclusions. Thirdly, the participants tested in this project were heterogeneous and we accepted all self-reports of hmTBI. We imposed no restrictions based on time since injury, number of mTBIs, nature of medical treatment received, or etiology. We argue that this ‘open-door’ policy would avoid cherry-picking and would add noise thereby making it more difficult to detect an effect of mTBI. Because we did not require medical verification of participants’ mTBI history it is possible someone would deliberately lie. This seems unlikely as there was the concurrent opportunity to serve as a control participant. This is different from the SR-mTBI participants who were recruited from UNR Athletics after mTBI diagnosis. The similar performance between the two mTBI groups was unexpected. It may be that their peak physical condition allows them to perform better than the typical subacute mTBI participant and that these data present an inflated level of post-mTBI WM ability. Regardless, these findings raise concerns that all mTBIs should be taken seriously. Furthermore, we acknowledge that better powered studies are needed to look at more subtle effects. Overall, these data highlight the need for continued rehabilitation approaches as they confirm the presence of lasting WM deficits long after an mTBI.

## Methods

### Participants

Table 1 provides demographic information for each experiment. Based on our empirically derived large effect size of group (Cohen’s d = 0.92) from our first paper. To preserve power (0.90), we needed 25 participants per group (G-Power)^47,83^. Participants self-reported their hmTBI by keypress response indicating if they had a hmTBI (‘Y’ or ‘N’). Participation did not depend on their mTBI history. Follow-up questions included reporting the number of mTBIs incurred, when the most recent took place, and the etiology of their mTBI (19 hx mTBI were SR-mTBI). Recent SR-mTBI participants were recruited from the athletic department and had medically diagnosed mTBI and were under active treatment. The Institutional Review Board at the University of Nevada, Reno approved all protocols. All participants provided written informed consent consent and were reimbursed $15/h or course bonus credit (their choice). All methods were carried out in accordance to the guidelines and regulations set by the University of Nevada, Reno ethics committee.

### Stimulus and procedure

#### Exp. 1A, Exp. 2. Change detection task

The task was presented on a 16″ MGC CRT monitor (75 Hz refresh rate, 1024 × 768) in MATLAB (The MathWorks, Natick, MA) using Psychophysics Toolbox 3.0 extension, using a Mac mini-1.4-GHz dual-core Intel Core i5. Participants were seated 57 cm from the display and instructed to maintain fixation throughout. Stimuli were presented in two rectangular areas subtending 7.1° × 12.2° of visual angle centered 4.6° from the fixation cross on a uniform medium gray background. Each trial, six colored squares (0.7° × 0.7°) were drawn from a set (cyan, white, red, blue, yellow, green, magenta). Stimuli were briefly presented symmetrically in each visual hemifield with a probe item appearing after a delay (Fig. 1A). Participants indicated whether the encoded stimulus and probe item matched. If no response was registered, the trial was considered incorrect. Trials were self-paced. Participants completed 24 practice and 120 experimental trials.

#### Exp. 1A. 3-Back task

The task was presented on a 24″ LCD monitor (Dell 1707 FPc,) using an Intel Core i7 CPU 2.93 GHz running E-Prime v2.0 (Psychology Software Tools, PA, USA; https://pstnet.com/e-prime-publications/). Participants completed three 3-back WM blocks using different task type (shapes, spatial, verbal; Fig. 1B) and randomized across participants. During the *spatial* 3-back task, participants maintained the location of green circles (3° visual angle, 500 ms) appearing sequentially in one of nine locations (followed by an inter-stimulus interval, 3000 ms). The *shapes* block using symmetrical novel polygons^84^, the *verbal* block used 20 consonants (Palatino size 30). The button presses, trial count, and timing of the task matched the spatial task. Participants pressed ‘J’ when the stimulus matched the item presented three items earlier; they pressed ‘F’ if they did not match. Participants completed 45 practice and 120 experimental trials (66% non-target, 7 min).

#### Exp. 1B. Auditory WM 3-back

Participants completed a 3-back WM task while hearing letters (consonants) or numbers (digits 1–9) spoken in separate blocks. Block order and response inputs were counterbalanced. The task was presented on a 16-in. MGC CRT monitor (75 Hz refresh rate, 1024 × 768) in MATLAB (The MathWorks, Natick, MA) with Psychophysics Toolbox 3.0 extension, using a Mac mini-1.4-GHz dual-core Intel Core i5.

#### Exp. 2. Methods

We collected behavioral, neuropsychological, and rsEEG data from participants who self-reported a hmTBI. We also collected data from SR-mTBI participants were recently (< 3 months) injured college-aged students. The inclusion of the subacute sample serves to clarify the relative impairment experienced by the hmTBI group. It also provided a comparison group in examining the time course of connectivity changes after an mTBI. We were interested in seeing if connectivity measures could be sensitive to detecting the cognitive and neural deficits in subacute and those with a hmTBI. We hypothesized that there would be more severely impaired performance and worse connectivity in the subacute mTBI group compared to those with a hmTBI; but that both would be worse than the control group. We predicted more severely abnormal rsEEG measures in the subacute mTBI participants compared to the group with a hmTBI.

### Cognitive testing

Participants completed several tests to measure cognitive performance, verbal learning, and attention. Tests included: Repeated Battery for the Assessment of Neuropsychological Status (RBANS)^85^, Trail Making Test Part B (TMT-B)^86^, California Verbal Learning Test-II (CVLT-II)^87^-Short, and the Psychomotor Vigilance Test (PVT)^88^.

#### rsEEG

EEG recordings were collected over 6 intervals of 3 min each, alternating between eyes open and closed (not analyzed)^89^. Participants remained still and maintained neutral thoughts. During eyes-open blocks, participants-maintained fixation. Blocks ended with a 1000 Hz tone. EEG data was recorded (1000 Hz, 256 high-impedance electrodes in a HydroCel Geodesic Sensor Net, vertex reference, Net Amps 300 amplifier, Net Station 4.5.5 EGI, Eugene, OR), running on a 2.7 GHz dual-core Apple Power Mac G5.

### EEG processing and analysis

Offline preprocessing (EEGLAB v14.1.2^90^ and ERPLAB v8.01^91^) included band-pass filtering (1–100 Hz), segmentation from block onset—180 s, and downsampled (10–10 international electrode system, 70 electrode channels) to reduce highly correlated signals. Independent components analysis (ICA, SOBI) removed artifacts without removing trials^92,93^. For consistency, we automated the process using Multiple Artifact Rejection Algorithm^94,95^. Subsequent analyses were done in Fieldtrip^96^ separately in the delta (1–5 Hz), theta (5–8 Hz), alpha (8–12 Hz), beta (12–30 Hz), and low gamma (30–80 Hz) bands^97–99^.

#### rsEEG analyses

We used the following measures: weighted phase lag index^100,101^, modularity^102^, local and global efficiency^102–104^, and network cost^105^. RsEEG data was analyzed adopting prior methods deemed successful in clinical populations^106^. These values measure synchronization, within network connections, neighboring and node pair interactions, and node-pair interactions, respectively.

##### Weighted Phase Lag Index (wPLI)

WPLI is a measure of synchronization that address issues of volume conduction in the assessment of connectivity measures in EEG^100,101^. WPLI allows for the measurement of coherence that does not rely on correlation or partial correlations. WPLI weights the cross spectrum based on magnitude of the imaginary component. This allows for limits in the influence of cross spectrum elements around the real axes. Overall, wPLI measures asymmetry in the distribution of the phase differences from instantaneous phases of the two-time series. The wPLI contribution of the observed phase leads and lags is weighted by the magnitude of the imaginary component of the cross-spectrum. WPLI is defined as follows^107,108^:

$$\mathrm{wPLI }\equiv \frac{|\mathrm{ E }\{\mathfrak{I}\{\mathrm{X}\}\}|}{|\mathrm{ E }\{\mathfrak{I}\{\mathrm{X}\}\}|}=\frac{|\mathrm{E}\{|\mathfrak{I}\{\mathrm{X}\}|\mathrm{sgn}(\mathfrak{I}\{\mathrm{X}\})\}|}{\mathrm{E}\{|\mathfrak{I}\{\mathrm{X}\}|\}}$$

The resulting WPLI is an absolute value between 0 and 1, such that 0 is the random phase difference with minimal strength of connectivity and 1 is the constant phase difference with maximum strength of connectivity^109^. $\mathrm{E}\{|\mathfrak{I}\{\mathrm{X}\}|\}$ denotes the imaginary and real component of the cross-spectrum. The wPLI was calculated using FieldTrip and was calculated for each electrode pair for every participant which created a wPLI matrix (70 electrodes × 70 electrodes) with wPLI values for each cell.

##### Graph theoretical metrics

In EEG connectivity, graph theoretical methods depict vertices and edges represent electrodes and connectivity strengths. We used the Brain Connectivity Toolbox (BCT)^104^ for Matlab.

##### Modularity

Modularity (Q) is the number of within-network module connections to all within-network module connections^102,104^ reflecting the balance of local versus broader interactions^110,111^. Modularity is measured from 0 (integration) to 1 (segregation). Integration across modules allows for global communication whereas segregation benefits local processing^103^. Modularity (Q) is defined by this equation^104^:

$$Q= \sum_{u\epsilon M}\left[{e}_{uu}- {\left(\sum_{v\epsilon M}{e}_{uv}\right)}^{2}\right]$$

Each network is subdivided into a set of non-overlapping modules M, and ${e}_{uv}$ is the proportion of all links that connect nodes in module $u$ with nodes in module $v$^104,112^.

##### Global efficiency

Global efficiency measures information transfer among node-pairs to clarify integration within a neural network^104,113^. Global efficiency is calculated as follows^104,113^:

$$Eglob= \frac{1}{n}\sum_{i\epsilon N}{E}_{i}= \frac{1}{n} \sum_{i\epsilon M}\frac{\sum j\in N,j\ne i {d}_{ij}^{-1} }{n-1}$$

here, ${E}_{i}$ is the efficiency of node $i$^104,113^ and *d*_*ij*_ is the shortest path between nodes $i$ and $j$.

##### Local efficiency

Local efficiency quantifies network segregation which measures information transfer among neighboring nodes^102–104,113^. It is calculated as follows^104^:

$$Eloc= \frac{1}{n}\sum_{i\epsilon N}{E}_{loc,i} = \frac{1}{n}\sum_{i\epsilon N}\frac{{{}_{j, h\epsilon N, j\ne i} {a}_{ij}{a}_{ih}[{d}_{jh}\left({N}_{i}\right)]}^{-1}}{{k}_{i}({k}_{i}-1)}$$

where the ${E}_{loc,i}$ is the local efficiency node $i$, and ${d}_{jh}\left({N}_{i}\right)$ is the length of the shortest path between $j$ and $h$, that contains neighbors of $i$^104^.

##### Network cost

The analyses were conducted at network costs ranging from 10 to 30%, step size 5%, to ensure that results were not due to specific threshold values^105^. The range of thresholds was based on those values that have previously produced graphs with small world characteristics^105^.

#### Exp. 3. RsfMRI

In Experiment 3 we collected rsfMRI data from the hmTBI group tested in Experiment 2. We were interested in identifying reduced connectivity measured by rsfMRI in the hmTBI group. All but two of the hmTBI cohort from Experiment 2 (see Table 1) provided rsfMRI data. The control group was taken from a prior rsfMRI study in participants reporting no history of head-injury, psychiatric or neurological conditions^114^. Participants received $50/h or course bonus credit.

##### fMRI methods and parameters

Participants completed 3 rsfMRI runs (5.3 min each). Participants closed their eyes, relaxed and maintained neutral thoughts. Functional images were acquired on a 3 T Philips (Andover, MA) MRI with an eight channel SENSE parallel head coil. A set of 155 T2^*^-weighted volumes were obtained (TR = 2000 ms, TE = 30 ms, 32 slices per volumes, slice thickness = 3 mm, FOV = 240 mm, matrix size 128 × 128). Data were aligned to a high-resolution 3D structural dataset using an echo-planar 3D T1-weighted image.

##### fMRI preprocessing

Preprocessing used AFNI^115^ (http://afni.nimh.nih.gov/afni/), SUMA^116^; http://afni.nimh.nih.gov/afni/suma/), and FreeSurfer^117,118^ (http://surfer.nmr.mgh.harvard.edu/) [*afni_proc.py* (http://afni.nimh.nih.gov/pub/dist/doc/program_help/afni_proc.py.html)]. The first two TRs were removed, data were despiked, slice-time and motion corrected, and spatially normalized to the MNI template. The data were bandpass filtered (0.01–0.2 Hz). Censoring relied on motion parameters and signal outliers^119,120^. Six motion parameter estimates, ventricular and white matter signals, and baseline, linear, quadratic, and cubic trends were removed by linear regression^121^.

##### Regions of interest and seeds

Neurosynth (neurosynth.org) was used to identify commonly used seeds for the networks of interest based on prior research indicating interactions between functional connectivity and WM performance; see Table 3^61,62,67–69,122–126^. Primary seed regions of interest (5 mm spheres, 19 voxels; rPCC, rDLPFC, rIPS, rAI) and secondary brain regions within the network (4 ROIs; 5 mm spheres, 19 voxels; vmPFC, rPPC, FEF, BA 47) were generated manually using AFNI.

**Table 3:** ROI seed locations.

Network	Seed locations	MNI (X, Y, Z)
DMN	rPCC, vmPFC	(5, − 49, 40), (− 4, 54, 0)
CEN	rDLPFC, rPPC	(44, 36, 20), (42, − 56, 50)
DAN	rIPS, FEF	(39, − 42, 51), (− 26, 10, 51)
SN	rAI, BA 47	(42, 0, 2), (38, 24, − 12)

All seed locations were selected using prior rsfMRI research that used WM as a behavioral correlate of connectivity.

##### Resting state analysis

To examine functional connectivity, we first evaluated connectivity between the time series data of the seed region and the rest of the brain (e.g., rPCC-Whole Brain). Additionally, we selected a second brain region within the selected network and correlated the time series between the two seed regions as a measure of network connectivity. AFNI’s *3dUndump* created the ROI from the specified coordinates. *3dmaskave* generated the time course in the seed region. *3dfim*+ correlated time courses within the seed regions and the whole brain and the primary seed region-second brain region to generate connectivity maps of Pearson’s r values. To normalize the r values, we converted them to z-scores using Fisher’s r-to-z transformation and the expression ‘*log*((1 + *a*)/(1 − *a*))/2’. We then compared the mean z-scores for each group.
